# Two-port access for laparoscopic surgery for endometrial cancer using conventional laparoscopic instruments

**DOI:** 10.1038/s41598-020-79886-8

**Published:** 2021-01-12

**Authors:** Kuan-Ju Huang, Ying-Xuan Li, Bor-Ching Sheu, Wen-Chun Chang

**Affiliations:** 1 Department of Obstetrics and Gynecology, National Taiwan University Hospital, National Taiwan University College of Medicine, 7 Chung-Shan South Road, Taipei, Taiwan; 2 Graduate Institute of Clinical Medicine, National Taiwan University College of Medicine, Taipei, Taiwan

**Keywords:** Oncology, Cancer, Surgical oncology, Techniques and instrumentation, Health care economics, Gynaecological cancer, Endometrial cancer

## Abstract

Minimally invasive surgery is the first-line management for endometrial cancer. The role of 2-port access laparoscopy (TPA) has been underestimated. Compared to conventional laparoscopic surgery (CL), TPA is associated with smaller total incision size and less postoperative pain. Compared to single-incision laparoscopic surgery, no specific instruments and surgical techniques are needed. This study primarily evaluated the surgical and pathologic outcomes of TPA with conventional instruments, and additionally evaluated the surgeon’s learning curve. Consecutive patients who underwent TPA and CL for endometrial cancer between 2015 and 2019 were included. Baseline characteristics were recorded. In total, 148 patients (TPA, 89; CL, 59) were identified. The baseline characteristics were similar, except for a greater proportion of patients in the CL group receiving para-aortic lymph node dissection (5.62% vs. 35.59%, *P* < 0.01). The mean operation time was significantly less in the TPA group (152.09 vs. 187.15 min; *P* < 0.01). Both the groups had comparable 5-year progression-free survival (TPA, 86.68%) and 5-year overall survival rates (TPA, 93.24%). Analysis of the learning curve showed that the operation time decreased after 3–4 procedures. TPA using conventional laparoscopic instruments for endometrial cancer is feasible and is easily accessible to patients and surgeons.

## Introduction

Uterine cancer is the most common gynecologic malignancy in the United States and in other developed countries including Taiwan^1,2^. Its incidence and mortality in Taiwan were 14.00 and 1.67 per 100,000 women in 2016, with 78.17% cases diagnosed at an early stage. Endometrial cancer (EC) accounts for 86.36% of all uterine cancers^1^. Minimally invasive total hysterectomy and bilateral salpingo-oophorectomy with or without lymphadenectomy is the first-line management for most newly diagnosed ECs^3,4^. Compared to laparotomy, minimally invasive surgery (MIS) has lower complication and blood transfusion rates and shorter hospital stay^5,6^. The Lap-2 study reported the feasibility and safety of conventional laparoscopy (CL) in staging uterine cancer^7^. Single-incision laparoscopic surgery (SILS) emerged as a result of improvements in instruments and camera technology and has been used in gynecologic malignancies; it provides better outcomes with regard to morbidity, postoperative pain, recovery period, and cosmetics^6,8^. However, the limitations and challenges with SILS, such as triangulation, instrument collision, and perspectives in surgical field, particularly in lymph node sampling, can render the technique challenging to surgeons and increase the operation time, which in turn poses a threat to patients because of prolonged anesthesia, a steep Trendelenburg position, and pneumoperitoneum^9–11^. Furthermore, the expensive instruments used in SILS may influence the patient’s decision-making regarding undergoing this procedure and limit the medical resources that the patient can afford.

In this study, we evaluated the surgical outcomes and cost for patients undergoing 2-port access (TPA) procedures using CL instruments for EC. In addition, we evaluated the learning curve associated with this technique over an expanded sample size.

## Materials and methods

This retrospective, single-institution study was approved by the National Taiwan University Hospital Research Ethics Committee (201908025RINA) and complies with the Declaration of Helsinki. A waiver of informed consent was obtained from the National Taiwan University Hospital Research Ethics Committee. Consecutive patients who underwent TPA and CL for EC from 2015 through 2019 at National Taiwan University Hospital were identified and included in the study. We had only included patients with clinical stage I–II uterine cancer. Patients who received sentinel lymph node biopsy (SLNB) were excluded. The baseline characteristics including age, body mass index (BMI), parity, medical history, surgical history, CA-125 level, and estimated uterine volume were recorded. Two surgeons (Dr. CW Chang and BC Sheu) performed surgical staging via TPA. Both of them had performed various conventional laparoscopic surgeries and TPA laparoscopic surgeries in the gynecology field^12–15^. In total, 8 surgeons (including Dr. CW Chang and BC Sheu) performed surgical staging. Operator experience was classified into 2 groups based on the number of surgical stagings conducted via MIS per year: > 10 and < 10. These surgeries were assisted by senior residents and junior residents for assistance with endoscope control and the uterine elevator, respectively. Surgical staging was conducted following the National Comprehensive Cancer Network guidelines, including hysterectomy, bilateral salpingo-oophorectomy (BSO), bilateral pelvic lymph nodes dissection (BPLND), and peritoneal washing cytology. Para-aortic lymph node dissection (PALND) was not performed routinely because of the associated minor risk of aortic metastasis in early-stage EC^16^. Omental biopsy was performed in selected patients^16^.

The surgical outcomes compared included operation time, blood loss, units of transfusion, hospital stay, pain score at 24 h, pain score at 48 h postoperatively, self-paid cost, total cost, and complication rates. The pathologic outcomes compared included lymph node yield, histology type, percentage of lymphovascular invasions (LVSI), stage cytology, adjuvant therapy, progression-free survival (PFS), and overall survival (OS). The operation time was defined as the interval between the initial skin incision and its closure. Blood loss was calculated as the volume of aspirated fluid in the bottle. Complications were defined as events requiring active treatment or prolonged hospital stay. Adjuvant chemotherapy was administered according to the National Comprehensive Cancer Network guidelines or recommendations of the multi-disciplinary tumor board and considered the patients’ age and pathologic risk factors including FIGO stage, histologic grade, depth of invasion, and lymphovascular invasion^16^.

### Surgical techniques

**Figure 1:**
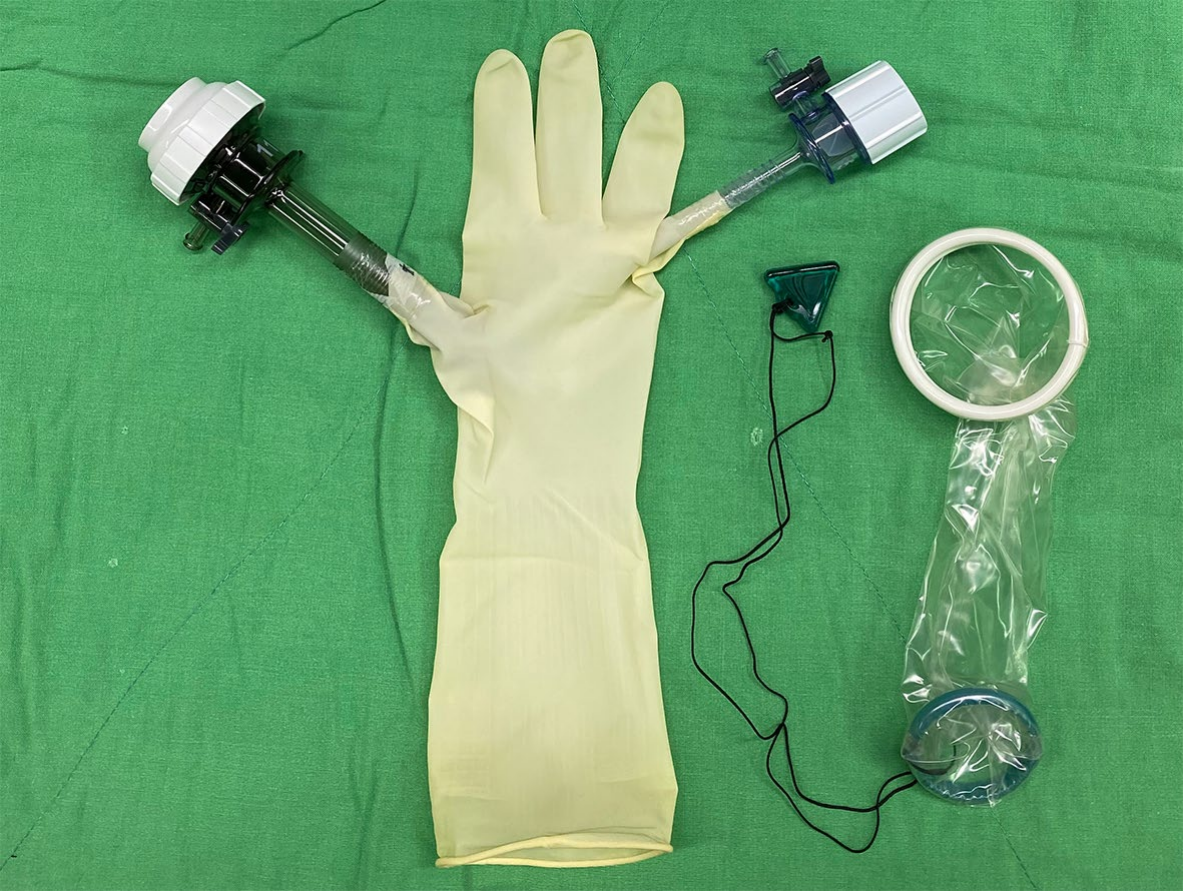
Umbilical port setting.

**Figure 2:**
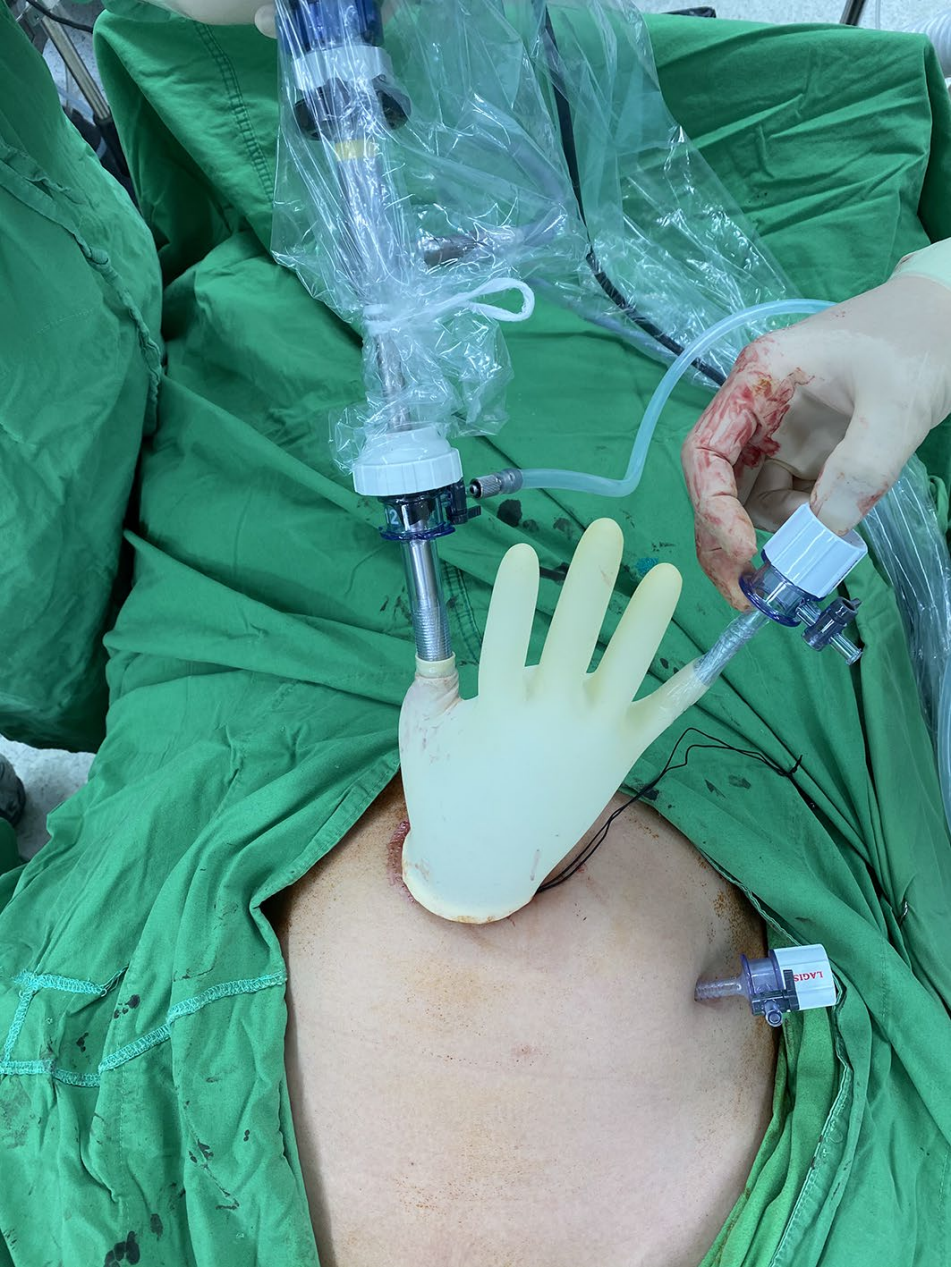
2-port access laparoscopic surgery.

With the patient under anesthesia, we placed the uterine elevator. A vertical 2-cm umbilical incision was made; the Alexis wound retractor (sized XS; Applied Medical, CA) was inserted, and a surgical glove was connected to the wound retractor. A 10-mm trocar and a 5-mm trocar were fixed to the fingertip portions with 3 M tape (Fig. 1). Another 5-mm trocar was inserted in the left lower abdomen (Fig. 2). In some cases, a third 5-mm port was created in the right lower abdomen. CO_2_ was insufflated through the 5-mm umbilical trocar to maintain an intra-abdominal pressure of 12 mm Hg. A rigid 10-mm 0-degree endoscope was used. The instruments for laparoscopic surgery included a standard rigid atraumatic grasper, a pair of scissors, a suction-irrigation system, and an electrosurgical system (LigaSure 5 mm Maryland jaw 37 cm sealer and divider, LF1737; Covidien, ValleyLab, Boulder, CO, USA). The surgeon stood to the patient’s left, and an assistant surgeon stood to the patient’s right and manipulated the rigid scope through the 10-mm umbilical port using his/her left hand.

**Figure 3:**
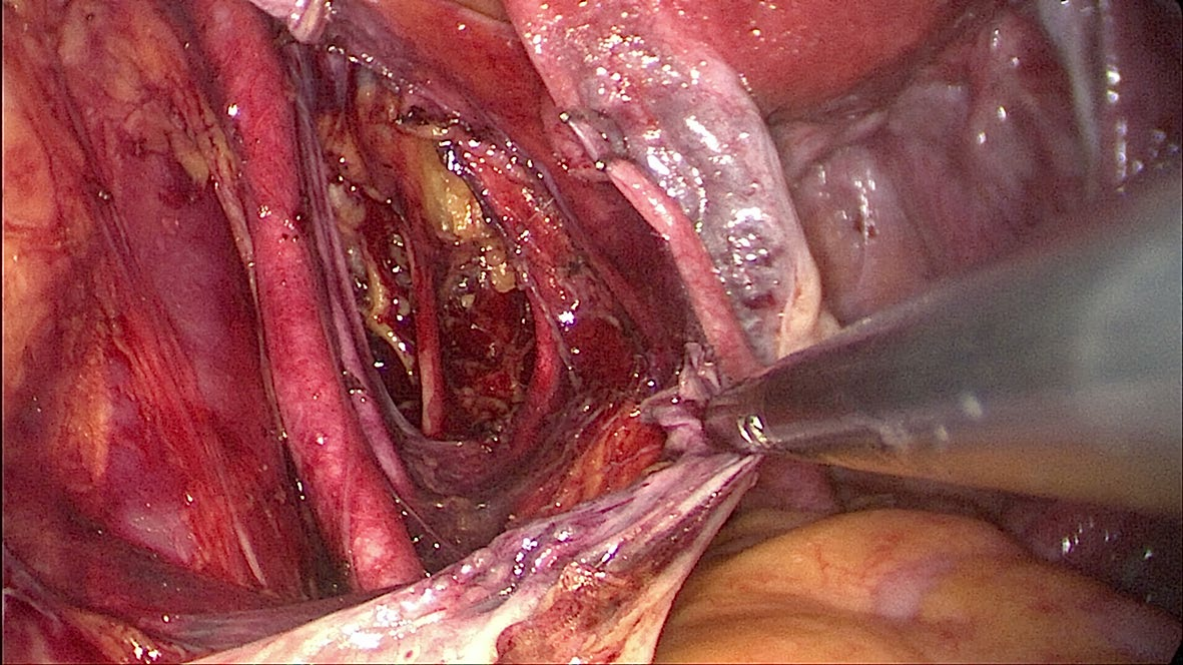
Dissection of the left pelvic lymph nodes.

**Figure 4:**
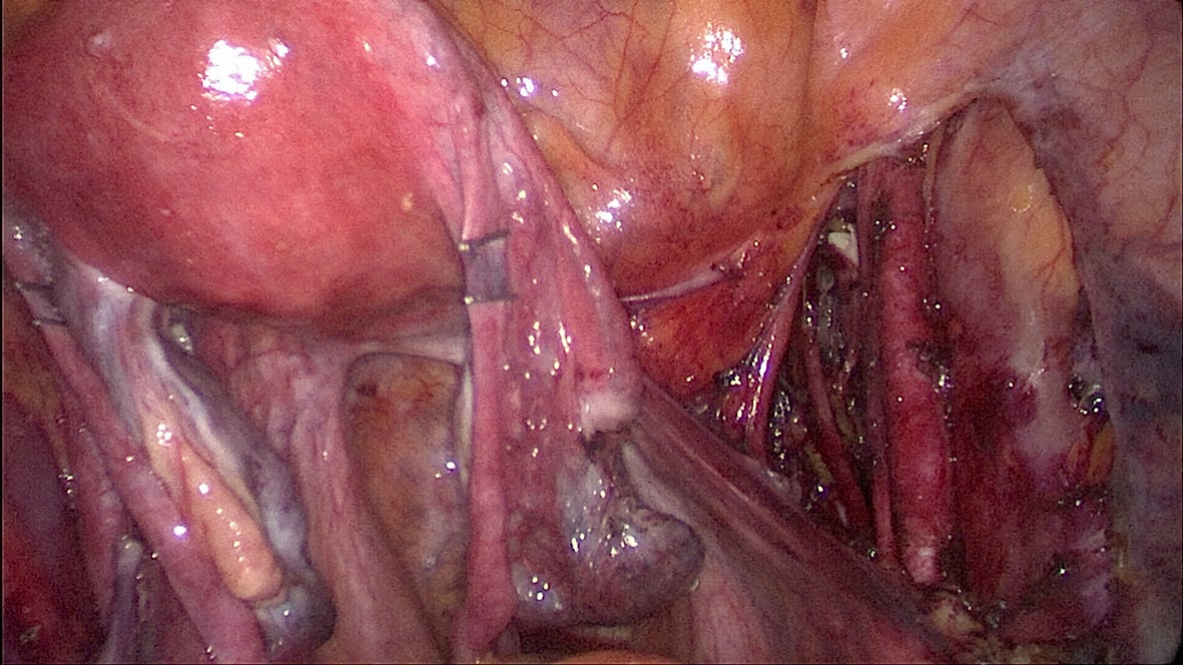
Dissection of the right pelvic lymph nodes.

The procedures started with peritoneal washing cytology with a Nelaton catheter. After ligation of the round ligament and infundibulopelvic ligament at one side, the external iliac and the obturator lymph nodes were removed by dissection with the LigaSure system (Figs. 3, 4). The same steps were performed on the other side. The specimens were placed in a cut fingertip of the surgical glove and removed through the fingertip of the surgical glove through the umbilical incision. Subsequently, the bilateral uterine arteries were clamped and coagulated using LigaSure. Diluted vasopressin (20 U/mL diluted in 100 mL sterile saline) was injected into the serosa of the vesicouterine junction for hydro-dissection. At the end of the laparoscopic procedure, we made an incision into the vesicouterine peritoneum with a pair of scissors to facilitate the entry of the anterior cul-de-sac during subsequent hysterectomy at the vaginal phase. Hysterectomy was completed transvaginally, and the vaginal cuff was closed with 2-0 Vicryl sutures. Omental biopsy was performed using LigaSure in selected patients. Some self-paid supplements, for example anti-adhesive barrier with BarriGel (HANBIO, TW) or Hyalobarrier (Anika Therapeutics, USA), and hemostatic agent TISSEEL (Baxter AG, Austria), were used upon preoperative patient authorization. The ports were then removed, and the umbilical fascia was closed with 2-0 Polysorb sutures (glycolide-lactide copolymer; 5/8 circle (Covidien PLC, Dublin, Ireland). The skin incisions were approximated using DERMABOND Mini Topical Skin Adhesive (Ethicon Inc., USA).

### Statistical analysis

SAS (Version 9.4, SAS Institute, Cary, NC) was used for the statistical analyses. Baseline characteristics were analyzed by independent t test, chi-square test or fisher’s exact test. The operative outcomes were analyzed by linear regression model for continuous variables, and logistic regression model for category variables. The pathologic outcomes were analyzed by independent t test, chi-square test or fisher’s exact test. 5-year progression free survival and overall survival were calculated by log-rank test. The results were presented as means ± standard deviations. Qualitative data or categorical data were expressed as percentages. The cumulative sum (CUSUM) method is an objective statistical tool assessing operators’ performance during a series of procedures, and has been used in evaluating the learning curve of physicians performing various procedures from different subspecialties^17^. This method was used for both operators (28 cases and 47 cases, respectively).

## Results

Between 2015 and 2019, 148 patients received surgical staging via MIS: 89 by 2-port TPA and 59 by CL. Table 1 lists the baseline characteristics of the TPA and CL groups. The mean age (TPA vs. CL, 55.89 vs. 54.19 y; P = 0.28), BMI (TPA vs. CL, 24.56 vs. 24.97 kg/m^2^; *P* = 0.64), and uterine volume (TPA vs. CL, 129.19 vs. 111.51 cm^3^; *P* = 0.56) were comparable between groups. Operator experience did not differ significantly (TPA vs. CL, 51.69% vs. 40.68%; *P* = 0.19). Washing cytology (TPA vs. CL, 100% vs. 93.22%; *P* = 0.01) and pelvic adhesion (TPA vs. CL, 30.34% vs. 15.25%; *P* = 0.04) were performed more often in the TPA group, whereas PALND was performed more often the in CL group (TPA vs. CL, 5.62% vs. 35.59%, *P* < 0.01). Table 2 lists the operative outcomes. The TPA group had a shorter mean operation time (TPA vs. CL, 152.09 vs. 187.15 min; *P* < 0.01 before adjustment, *P* < 0.01 after adjustment) and less mean 48-h pain scores (TPA vs. CL, 2.5 vs. 2.74, *P* = 0.03 before adjustment, *P* = 0.04 after adjustment) but 39% higher self-paid costs (TPA vs. CL, 1645.43 vs. 1184.67 USD; *P* < 0.01 before adjustment, *P* = 0.02 after adjustment). Blood loss, transfusion units, hospital stay, 24-h pain scores, and total cost did not differ significantly between the groups.

**Table 1:** Baseline characteristics.

Characteristic	CL		TPA		*P* value
Mean (SD)	(n = 59)		(n = 89)		
**Preoperative**					
Age, years	54.19	(8.20)	55.89	(10.72)	0.28
BMI, kg/m^2^	24.97	(4.77)	24.56	(5.46)	0.64
Parity (%)	42	(71.19)	68	(76.40)	0.48
Medical history	0.56	(0.97)	0.84	(1.15)	0.12
Surgical history	0.46	(0.75)	0.31	(0.63)	0.21
CA-125, U/mL	27.63	(27.27)	31.02	(43.33)	0.61
Operator* (%)	24	(40.68)	46	(51.69)	0.19
Uterine volume, cm^3^	111.51	(83.16)	129.19	(94.55)	0.56
**Intraoperative**					
Omentectomy (%)	12	(20.34)	24	(26.97)	0.36
Washing cytology (%)	55	(93.22)	89	(100.00)	**0.01**
PALND (%)	21	(35.59)	5	(5.62)	** < .01**
Adhesion (%)	9	(15.25)	27	(30.34)	**0.04**

*P* < 0.05 is in bold.

*Percentage of surgeons performing < 10 cases of minimally invasive gynecological oncology surgeries per year.

**Table 2:** Operative outcomes.

Outcome	CL		TPA		Univariate model		Multivariate model	
Mean (SD)	(n = 59)		(n = 89)		Β (95% CI)	*P* value	Β (95% CI)	*P* value
Operative time, min	187.15	(41.87)	152.09	(44.26)	− 35.06 (− 49.44~ − 20.69)	** < .01**	− 26.63 (− 42.39~ − 10.88)	**0.01**
Blood loss, mL	99.66	(129.87)	77.30	(99.39)	− 22.36 (− 59.68~14.97)	0.24	− 13.97 (− 55.38~27.43)	0.51
Transfusion unit	0.10	(0.44)	0.07	(0.36)	− 0.03 (− 0.17~0.10)	0.61	0.02 (− 0.12~0.16)	0.81
Hospital stay, days	3.81	(2.40)	3.85	(1.36)	0.04 (− 0.57~0.65)	0.90	0.20 (− 0.47~0.87)	0.56
Pain score (24H)	3.42	(0.86)	3.18	(0.84)	− 0.24 (− 0.52~0.04)	0.09	− 0.25 (− 0.57~0.07)	0.12
Pain score (48H)	2.74	(0.76)	2.50	(0.55)	− 0.24 (− 0.46~− 0.03)	**0.03**	− 0.25 (− 0.50~− 0.01)	**0.04**
Self-paid Cost, USD*	1184.67	(756.35)	1645.43	(680.08)	460.76 (161.64~759.88)	** < .01**	407.54 (62.20~752.89)	**0.02**
Total Cost, USD*	4860.15	(878.97)	5150.76	(747.46)	290.61 (− 45.07~626.29)	0.09	310.42 (− 71.19~642.03)	0.11
Complication** (%)	3	(5.08)	2	(2.25)	1.54 (1.07 − 14.15)	0.36	2.34 (1.09 − 3232.46)	0.89

*P* < 0.05 is in bold.

*Estimated by 30.0 New Taiwan dollars to 1 USD.

**OR estimated by logistic regression model.

Table 3 lists the pathologic outcomes. Lymph node yield (TPA vs. CL, 7.95% vs. 7.21%, *P* = 0.78), histology (grade 1 endometrioid type; TPA vs. CL, 81.40% vs. 88.68%, *P* = 0.52), and LVSI (TPA vs. CL, 14.77% vs. 20.34%, *P* = 0.38) were similar across the groups. Stage IA cases accounted for 78.65% and 83.05% cases in the TPA and CL groups, respectively (*P* = 0.16). Cytology was positive in 21.35% patients in the TPA versus 23.64% in the CL group (*P* = 0.66). Adjuvant therapy was administered to 34.46% of patients in the TPA group and 20.34% of patients in the CL group (*P* = 0.14).

**Table 3:** Pathologic outcomes.

Outcome	CL		TRA		*P* value
Mean (SD)	(n = 59)		(n = 89)		
Lymph node yield	15.00	(7.21)	14.64	(7.95)	0.78
**Histology (%)**					0.52
Low grade	47	(88.68)	70	(81.40)	
High grade	5	(9.43)	13	(15.12)	
CSM	1	(1.89)	3	(3.49)	
LVSI (%)	12	(20.34)	13	(14.77)	0.38
**Stage (%)**					0.16
IA	49	(83.05)	70	(78.65)	
IB	7	(11.86)	8	(8.99)	
II	0	(0.00)	8	(8.99)	
IIIA	2	(3.39)	1	(1.12)	
IIIC1	1	(1.69)	2	(2.25)	
Positive cytology (%)	13	(23.64)	19	(21.35)	0.66
Adjuvant therapy (%)	12	(20.34)	28	(31.46)	0.14

The 5-year PFS (TPA vs. CL, 86.68% vs. 94.06%; *P* = 0.31) and 5-year OS (TPA vs. CL, 93.24% vs. 96.03%; *P* = 0.64) did not differ significantly between the groups. The 5-year PFS (TPA vs. CL, 90.85% vs. 94.03%; *P* = 0.76) and 5-year OS (TPA vs. CL, 96.59% vs. 95.99%; *P* = 0.78) did not differ even after excluding patients with carcinosarcoma (Table 4).

**Table 4:** Progression-free survival and overall survival.

	CL		TRA		Log-rank test
	(n = 59)		(n = 89)		*P* value
**Progression-free survival (%, 95% CI)**					0.31
Year 2	96.25	(89.54–99.63)	92.01	(84.77–97.06)	
Year 3	94.06	(85.92–98.85)	90.01	(81.74–95.99)	
Year 5	94.06	(85.92–98.85)	86.68	(75.95–94.59)	
**Overall survival (%, 95% CI)**					0.64
Year 2	96.03	(88.94–99.61)	95.14	(88.42–99.06)	
Year 3	96.03	(88.94–99.61)	93.24	(85.46–98.19)	
Year 5	96.03	(88.94–99.61)	93.24	(85.46–98.19)	

**Figure 5:**
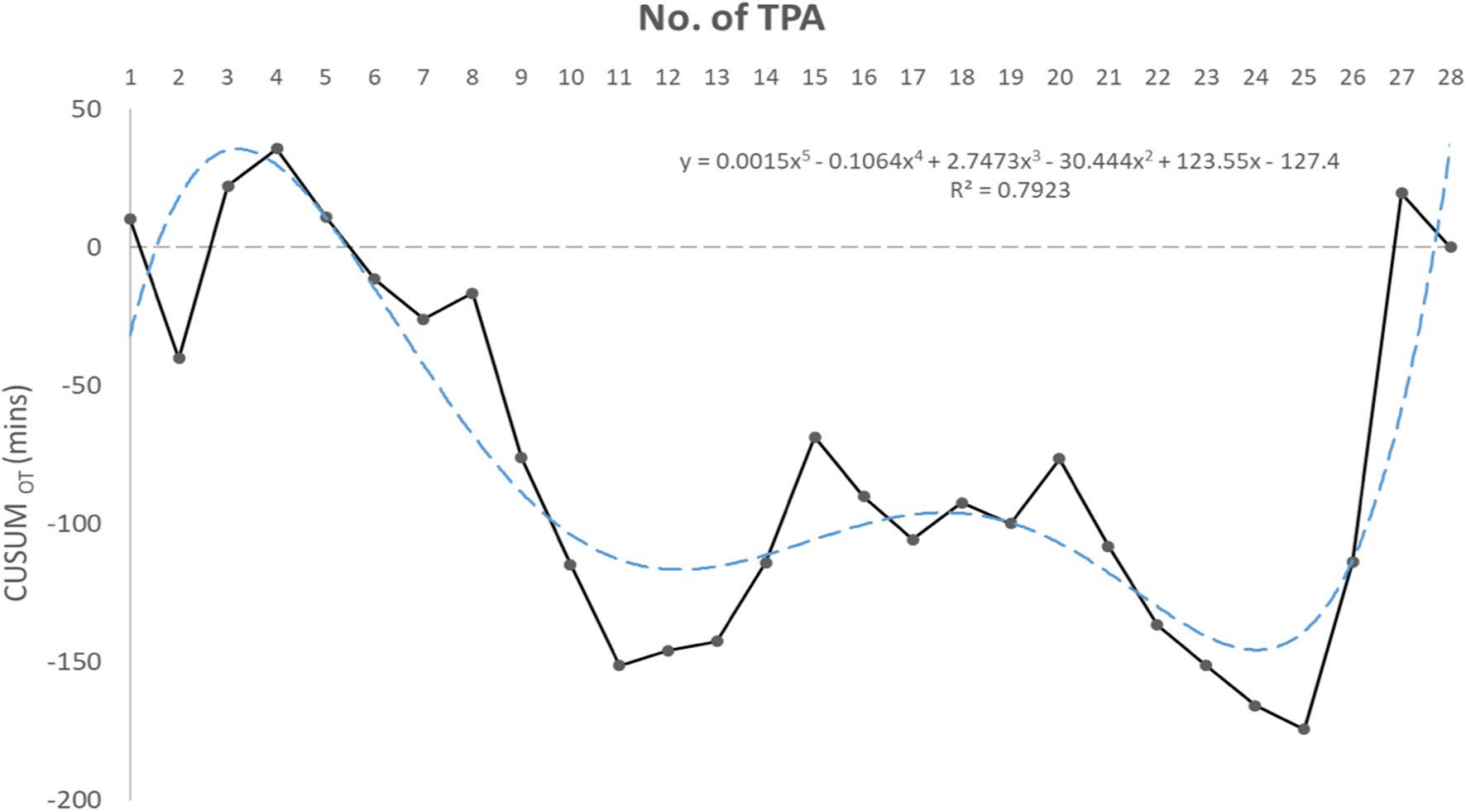
Learning curve for operator 1.

**Figure 6:**
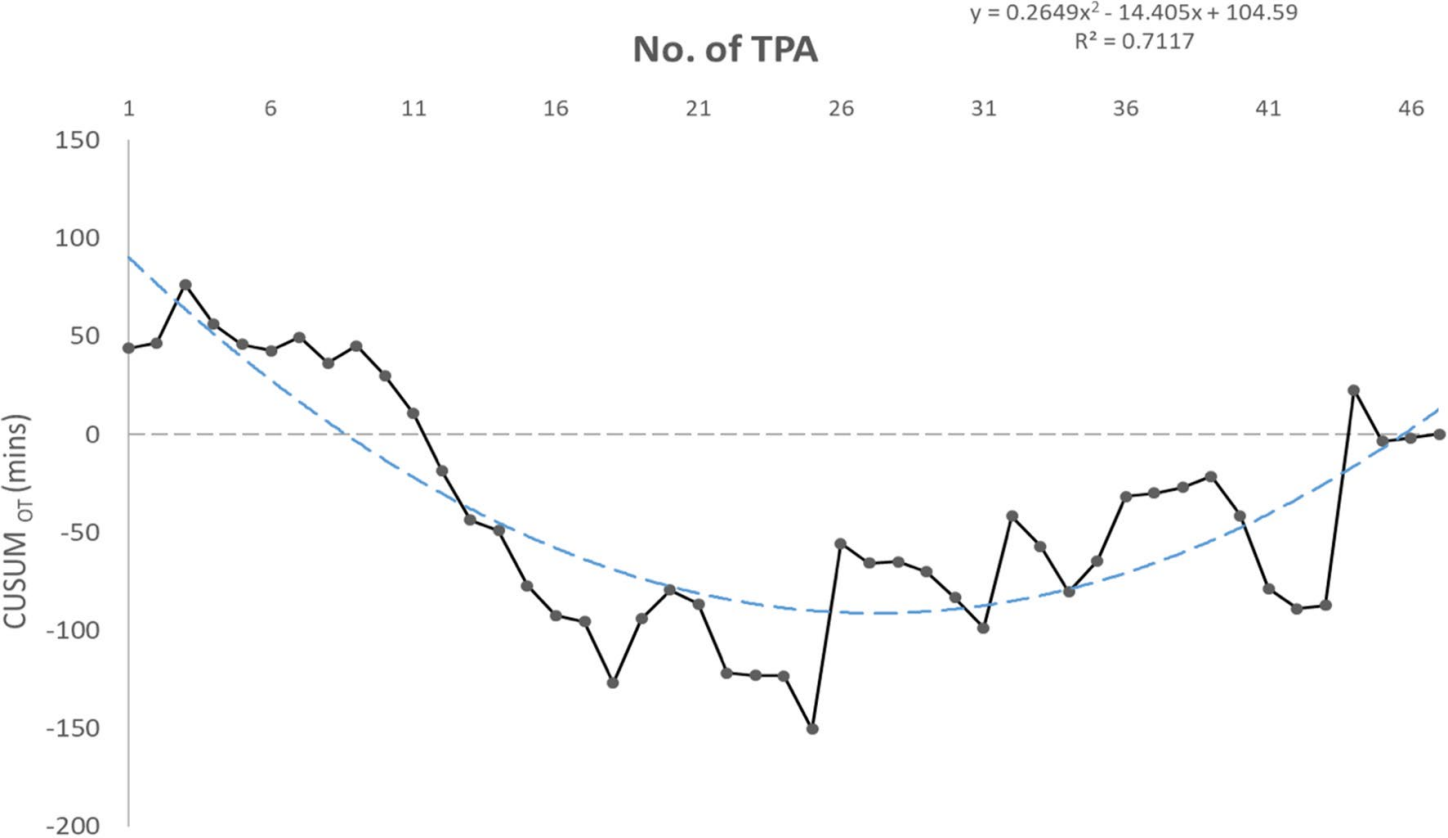
Learning curve for operator 2.

In learning curve analysis, the upward CUSUM chart for operator 1 and 2 were rising in the initial 4 and 3 procedures, suggesting the initial learning phase. The curves leveled off for the following procedures, with ongoing maintenance of competence for the next 15 procedures from the case 11th for operator 1, and 16 procedures from the case 16th for operator 2, respectively. (Figs. 5, 6). A trend toward increased operation time could be seen in the latter cases.

## Discussion

Laparoscopic surgery has recently become the mainstay of treatment for uterine cancer. The need for minimally invasive surgeries has resulted in the evolution of procedures within conventional laparoscopy, SILS, and robotic-assisted surgery. Across these, factors concerning surgical outcomes include operation time, blood loss, cosmetics, postoperative pain; however, survival- or relapse-related outcomes are comparable^18^. Furthermore, psychosocial factors, including cost, surgical technique, learning curve, and physician preference vary among these techniques. In the past, the role of TPA had been underestimated because it required more incisions than SILS and was less flexible than CL. In fact, the instruments used in TPA are easily accessible and are familiar to most surgeons. Via the 2-cm umbilical incision, the specimens can be retrieved relatively easily, and the assistant can focus on laparoscopic control. Compared to CL surgery, TPA has the benefits of smaller total wound size, less postoperative pain, and shorter hospital stay^11^. Furthermore, TPA allows for flexible triangulation, easy access, and reduced cost compared to SILS or robotic-assisted surgery^9,19^. In addition, TPA allows for easier insertion of the drain tube that is needed for postoperative monitoring in most cases, via its second 5-mm port. These benefits of TPA draw favor from patients and surgeons for surgical staging.

### Operation time was comparable for TPA and CL

A review of the relevant literature shows that the operation time ranges widely among surgical methods and procedures. Paek et al. first reported using TPA in 21 patients with EC in 2012, wherein 5 trocars were used in the surgery^11^. The operation time was significantly longer than with CL (238 vs. 188 min); however, TPA was associated with reduced postoperative pain and hospital stay (5 vs. 8 days). The author also compared SILS and CL in 2014; however, operation time remained shorter with CL (183 vs. 173 min)^20^. These results indicate that TPA requires a longer operation time than CL or SILS. However, PALND were performed in all patients, and TPA had higher yield than CL (13 vs. 5 lymph nodes) in the first study^11^. In the second study, PALND was performed in only 18.9% of the patients from both the groups, which makes direct comparison between TPA and SILS infeasible. We performed TPA with 3 trocars (4 trocars in selected cases), which resulted in a shorter operation time (152.09 min). This is likely because of the smaller BMI in our cohort, and we had not performed PALND. EC is usually associated with comorbidities such as obesity, metabolic syndrome, and advanced age. A prolonged operation time causes regional circulatory changes and increases the risk of deep vein thrombosis in these patients^10^. To complete surgical staging via SILS, precurved and flexible-tip instruments were used in most studies, which may add to the surgery cost. For CL, we use instruments that are readily available in most hospitals and are not as expensive as commercial port systems and tailored instruments for SILS. In their 2014 report, Fagotti et al. reported that the operation time for total hysterectomy BSO, and BPLND via SILS was 142 min, which increased to 192 min if PALND was performed^19^. Barnes et al. reported their results with SILS in 2017, and their operation time was 186 min with total hysterectomy, BSO, BPLND, and PALND^8^. The skill level of these experts must have significantly influenced their results. However, performing these procedures requires knowledge of specific instruments and an experienced assistant; hence, they are associated with a notable learning curve. Our results show that TPA is a feasible surgical option in EC for both surgeons and patients with reasonable operation times.

### Patients experienced less pain 48 h postoperatively with TPA

The 48-h pain scores for TPA and CL were 2.5 and 2.74 (*P* = 0.04), respectively. The favorable pain score is likely because of a smaller incision size and less incisions^11,18^. The larger umbilical port incision in TPA was negated by the reduced incision counts. Owing to less postoperative pain, patients could ambulate earlier, which prevented hypercoagulation and related complications.

### TPA is a safe approach for surgical staging of EC

The complication rates did not differ significantly between groups in our study. The recurrence rates were 7.99%, 9.99%, and 13.32% at 24, 36, and 60 months in this study. Galaal et al. conducted a Cochrane review in 2015 and reported the recurrence rates for laparoscopic surgery to be 8.6%, 11.4%, 12.6%, and 20% among studies with 38.5, 36, 44, and 84 month follow-ups, respectively^3^. The laparoscopic group of the LACE trial had 22.4% of the patients receive adjuvant therapy, with a recurrence rate of 17.2% at 4.5 y^21^. Another study comparing recurrence and survival among different minimally invasive surgeries with 34.2% of patients receiving adjuvant therapy also reported a recurrence rate of 7.3–9.9% at a median follow-up of 31.1–33 months. The study reported no differences among robotic surgery, CL, and SILS^18^. The author also found carcinosarcoma as an independent risk factor for early recurrence. In our TPA cohort, the recurrence rate was comparable between groups, in line with previous observations. Because 3 of our TPA patients (3.37%) had carcinosarcoma compared to only 1 CL patient (1.69%), we compared PFS and OS after excluding these patients. The differences were insignificant.

### TPA is an easy approach for surgeons

The upward CUSUM curves for operator 1 and 2 were rising in the initial 4 and 3 procedures, suggesting the initial learning phase. The curves leveled off for the following procedures, with ongoing maintenance of competence. In comparison, Barnes et al. evaluated SILS for EC and reported reduction of operation time after 80 cases (191–152 min) with at least 6 months of experience in SILS surgery before starting surgical staging^8^. The author advised that any surgeon intending to apply full uterine cancer staging should undergoes the general learning curve of SILS before adopting lymphadenectomy^8^. The complex technical requirements and instruments may make adopting this approach challenging. Our analysis suggests that for surgeons familiar with CL surgery in gynecologic oncology, performing TPA laparoscopic surgery using conventional laparoscopic instruments for EC is easy. The increased operation time seen in our latter cases could be because of the surgeons demonstrating the approach for teaching purposes.

### Patients were willing to spend more on self-paid costs

The self-paid cost was 39% higher for TPA; however, the total cost did not differ between groups (TPA vs. CL, 5150.73 vs. 4860.13 USD; *P* = 0.09). In Taiwan, the National Health Insurance covers 90% of the surgical fee. The patients pay the rest, including for antiadhesive barrier, hemostatic agents, specific surgical instruments, ward upgrades, and diets. Because the surgical instruments are similar between TPA and CL, the increased self-paid cost may be from the elective medical supplements, or patients’ choice of a better hospitalization experience and services.

To our knowledge, only few studies focused on the cost of TPA surgical staging. In this regard, we also compared TPA with CL over an expanded sample size. Recently, SLNB has evolved and has been considered as an alternative to pathologic staging^16^. Although we used this approach in some patients, we did not include those data given most previous studies comparing surgical methods did not include SLNB. A limitation of our study is that we cannot compare our results with SILS as we do not perform SILS at our facility. Robotic surgery, however, had similar operative outcomes for EC surgical staging although with a higher surgical cost^22^. However, in Taiwan, robotic surgery is not covered by National Health Insurance. During the study period, 8 patients received robotic-assisted surgical staging at our center; however, the sample size is insufficient for analysis.

In conclusion, this study shows that TPA using CL instruments is feasible and safe in patients with EC. This surgical approach has a shorter learning curve and uses instruments and equipment easily available at most hospitals. Furthermore, TPA costs less, improving patient affordability. Further large-scale studies comparing TPA with SILS and robotic-assisted surgery are needed.

## Supplementary information

Supplementary Information.
