# Supplementary material for: Two-port access for laparoscopic surgery for endometrial cancer using conventional laparoscopic instruments
Source: Sci Rep. 2021 Jan 12;11:615. doi: 10.1038/s41598-020-79886-8 (PMC7804851; doi:10.1038/s41598-020-79886-8)

**Two-port Access for Laparoscopic Surgery for Endometrial Cancer Using Conventional Laparoscopic Instruments**

Kuan-Ju Huang, MD^1^, Ying-Xuan Li, MD^1^, Bor-Ching Sheu, MD, PhD^1, 2^, Wen-Chun Chang, MD^1^*

1. Department of Obstetrics and Gynecology, National Taiwan University Hospital, National Taiwan University College of Medicine, Taipei

2. Graduate Institute of Clinical Medicine, National Taiwan University College of Medicine, Taipei

*****Corresponding Author: Wen-Chun Chang

**Figure Legends**

Supplementary Figure 1 The cut fingertip of a surgical glove is elastic, and around 1.5-2 cm in diameter.


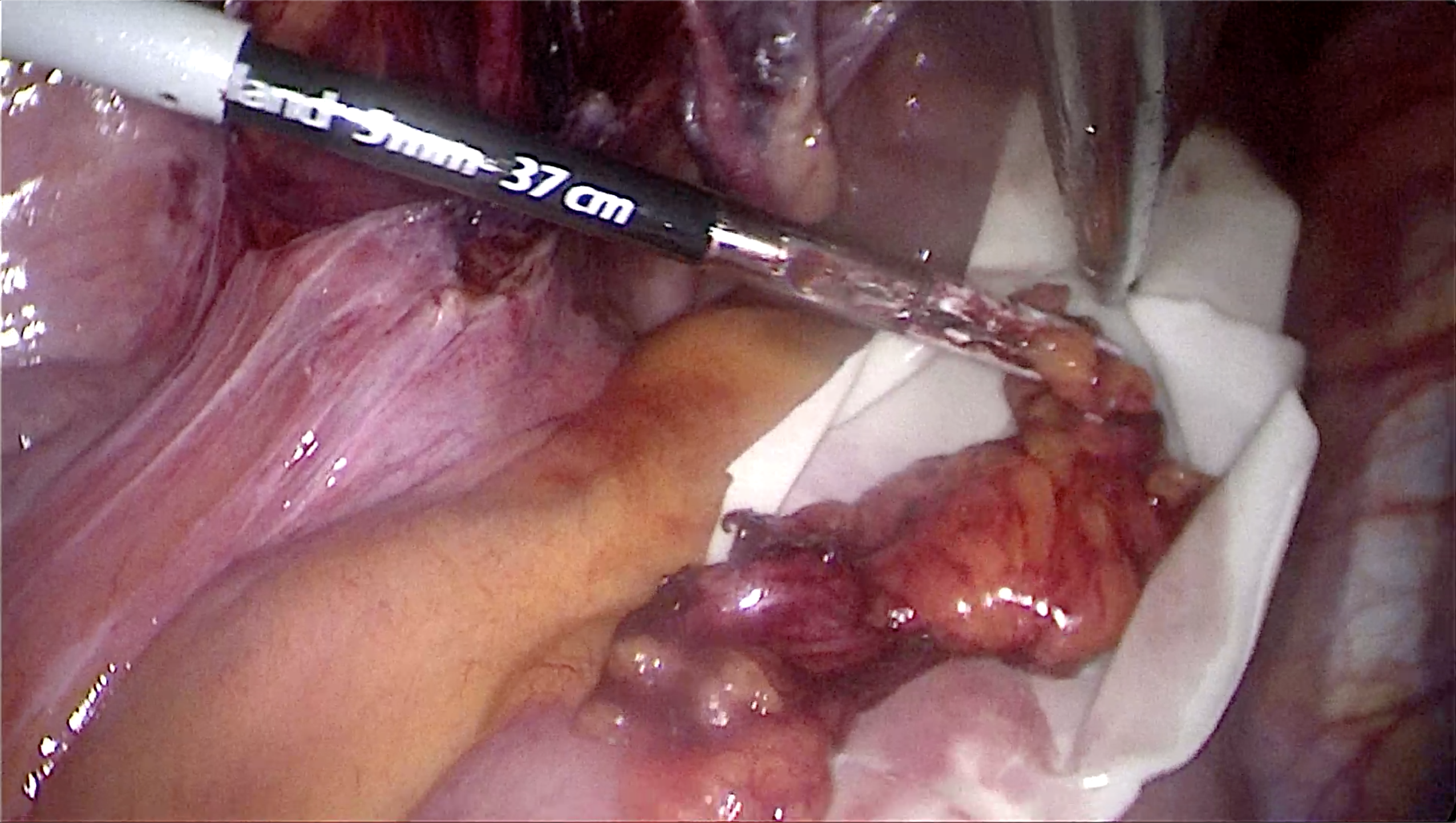


Supplementary Figure 2 The cut fingertip of a surgical glove can pass the umbilical wound (2 cm in size) easily.


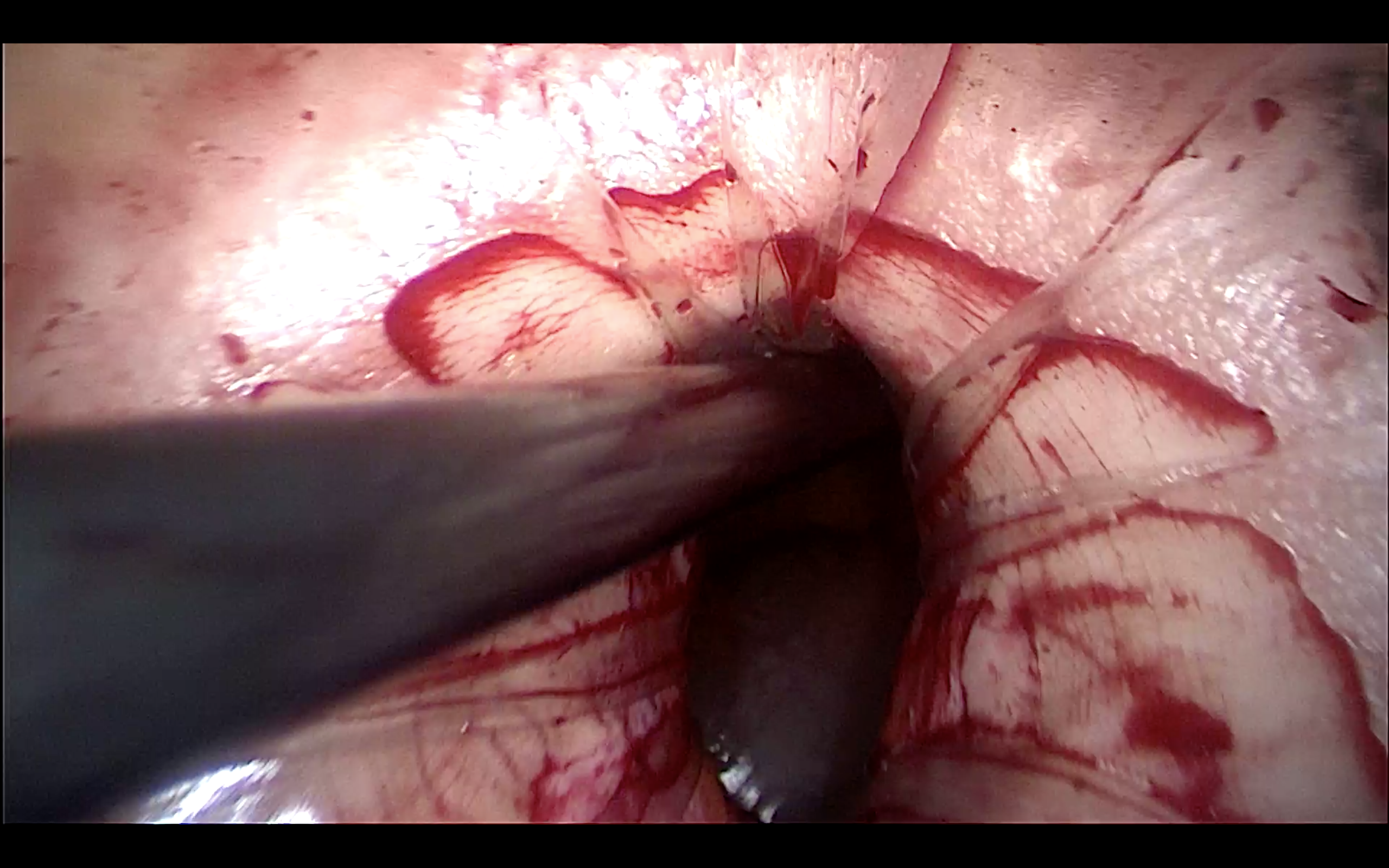

Supplement: Supplementary file 1 — Supplementary Information. [file 41598_2020_79886_MOESM1_ESM.docx]
